# Raiding nature’s genetic toolbox for UV-C resistance by functional metagenomics

**DOI:** 10.1038/s41598-024-83952-w

**Published:** 2025-01-02

**Authors:** Garrett A. Roberts Kingman, Justin L. Kipness, Lynn J. Rothschild

**Affiliations:** 1 NASA Postdoctoral Program in Astrobiology, Ames Research Center, Moffett Field, CA USA; 2 Department of Cell Biology & Biochemistry, Brown University, Providence, RI USA; 3 NASA Ames Research Center, Planetary Systems Branch, Moffett Field, CA USA

**Keywords:** Biotechnology, Ecology, Evolution, Microbiology

## Abstract

As we assess the habitability of other worlds, we are limited by being able to only study terrestrial life adapted to terrestrial conditions. The environments found on Earth, though tremendously diverse, do not approach the multitude of potentially habitable environments beyond Earth, and so limited terrestrial adaptive capabilities tell us little about the fundamental biochemical boundaries of life. One approach to this problem is to use experimental laboratory evolution to adapt microbes to these novel environmental conditions. This approach can be dramatically improved through functional metagenomics, large-scale introduction of foreign genetic material to screen for phenotypes in a new host organism. This takes advantage of Earth’s immense biological diversity with high-throughput screening for genetic tools that can facilitate adaptation. We address a key gap in functional metagenomics work by exploring the impact of the experimental parameters chosen for functional metagenomics libraries. Experimental design dictates both fragment size and copy number, and we show that both can have outsized effects on the resultant phenotypes in non-intuitive ways. These results highlight the potential of functional metagenomics for adapting life rapidly to challenging new environments, with important implications in both astrobiology and bioindustry, while also emphasizing the impacts of decisions in experimental design.

**Supplementary Information:**

The online version contains supplementary material available at 10.1038/s41598-024-83952-w.

## Introduction

A fundamental unanswered question of biology is “What are the environmental limits of life’s biochemistry and what adaptations help it approach these limits?” This question has critical applications ranging from astrobiology, where the answers can help us target our search for life beyond Earth, to bioindustry, where the answers can enable use of biological tools in chemically-driven environments that are biologically challenging. Notably, life exists nearly everywhere on Earth where liquid water is present^1,2^, which is at once a testament to its incredible evolutionary adaptability and a severe impediment to our attempts to understand its limits, as its limits are not readily apparent.

Many studies have examined the environmental limits of extant extremophiles^3^ and discovered incredible tolerance to heat^4^, cold^5^, salt^6^, nuclear^7,8^ and ultraviolet radiation^9,10^, and numerous additional stressors. However, such work, while exceptionally valuable, is fundamentally limited by only being able to study extant organisms that have evolved under environmental conditions found on the modern Earth (or have retained adaptations to earlier conditions). Therefore, their limits likely reflect the limited conditions of Earth rather than true biological constraints. Candidate locales for potential life beyond Earth include Martian perchlorate brines^11^ and the clouds of Venus^12^, both of which differ dramatically from everywhere on modern Earth, leaving us ignorant about their compatibility with life.

Similarly, many bioindustrial processes are also limited by tolerance to their imposed environments, such as chemicals introduced for biomass pretreatment in biofuel production from lignins^13^ and by the toxicity of the produced molecules^14,15^. Others seek to increase performance at either low temperatures for maximizing energy efficiency^16^ or high temperatures for maximizing reaction rates^17^. Large scale industrial biomining, though efficient, is limited in its deployment by the environmental tolerances of key microbes^18^.

Additionally, many of the currently known limits to life reflect the bounds of incidental tolerance to other stressors, rather than the result of direct evolutionary pressures. For example, the most perchlorate-tolerant organisms currently known can grow in concentrations in excess of 1 M^19,20^, despite the most concentrated perchlorate deposits known on Earth barely reaching parts per million^21–23^. Similarly, *Deinococcus radiodurans* can withstand 5,000 Gy of ionizing radiation with no discernible effect on viability^24^ even though the most naturally radioactive locale on Earth yields exposures of only 0.4 Gy per year^25^. Thus, these extreme resistances are likely incidental consequences of tolerance to salinity and desiccation^26^, respectively. Other known limits, including low temperature, pressure, and UV-C radiation (200–280 nm, which is considered fully blocked by the modern Earth’s atmosphere^27^) may likewise not represent the limit reached by evolutionary pressures. This suggests that in the presence of direct evolutionary pressures, these limits which reflect incidental tolerance could be further extended. Nonetheless, the genetic tools evolved to address these related environmental challenges provide an invaluable starting point if we can figure out how to identify and make use of them.

When studying these limits, even in a one-dimensional manner, for astrobiology or industrial applications, we face two fundamental limitations. First, we don’t know if this is a true limit, even using organisms fundamentally similar to our own, and in many cases like the above examples, we have strong reasons to believe it isn’t. Second, we only partially understand how they do it, with severe limitations on our ability to use these genetic tools for our own purposes or to enhance them further.

One approach to addressing these problems is through adaptive laboratory evolution, which applies selective pressures to enhance reproductive fitness under the chosen environmental conditions^28,29^. Chance mutations which increase fitness increase in frequency in the experimental population, which can accumulate additional beneficial mutations over time. This technique has been applied successfully in many contexts to favor the evolution of new capabilities, most famously the ability for *Escherichia coli* to metabolize citrate in the Lenski lab’s long term evolution experiment^30^, as well as many strains used for bioindustrial production^31,32^. Fitness increases of 50 to 100% in the first few months are common, followed by decreasing rates of gain^28^. However, despite its many successes, adaptive laboratory evolution is limited by the availability and evolvability of adaptive variants. For example, in the Lenski experiment, *E. coli* required 12 years and 31,000 generations to evolve the ability to metabolize citrate^30^, due to multiple required mutational steps that on their own yielded little to no fitness gains. Other desired traits may have equally complex genetic paths and so never be realized by adaptive laboratory evolution experiments despite being biologically possible. This is particularly likely to be the case when considering extraterrestrial environments unlike any on Earth.

Importantly, unlike axenic laboratory cultures, natural evolutionary processes do not occur in isolation and frequently utilize horizontal gene transfer to access molecular tools previously developed by other species^33–36^. Even distantly related species share sufficient molecular mechanisms to transcribe and translate foreign DNA, often acquired through highly mobile plasmids, viruses, and transposable elements^37,38^, which can lead to new capabilities such as perchlorate reduction^39^ and enhanced survival in extreme environments^40,41^. This suggests a similar experimental approach may likewise combine the best aspects of the two methods. Indeed, synthetic biology-based approaches utilizing exogenous genetic constructs selected from the literature have provided many dramatic illustrations of expanding an organism’s environmental tolerances, including increased resistance to desiccation^42^, salinity^43^, low temperatures^44^, and radiation^45^, as well as the ubiquitous use of antibiotic resistance as an essential tool for virtually all processes involving recombinant DNA. However, these genetic tools are typically identified only through laborious efforts^45^ that do not lend themselves to scalability or understanding more than a tiny fraction of what evolution has exquisitely crafted and honed over billions of years. Thus, while the introduction of foreign DNA provides a powerful and complementary mechanism to traditional genetic mutation to produce rapid and dramatic increases in fitness, its applications in synthetic biology are limited by insufficient knowledge of existing natural genetic resources.

Functional metagenomics is a technique to experimentally discover gene function by cloning random genetic sequences from a diverse range of species into a highly diverse library pool in a tractable laboratory organism such as *E. coli*. Isolates from this library, each containing a single genetic fragment, can be assayed individually or the entire pooled library can be subjected to adaptive laboratory evolution to select for any sequences that increase fitness. This latter approach attempts to replicate and extend the natural process of horizontal gene transfer to efficiently screen diverse taxa for transferable cassettes conferring resistance to extreme environments, taking advantage of our greatest natural resource: Earth’s tremendous biodiversity. Unlike targeted synthetic biology design, it is not biased towards already known genes or pathways, and does not even depend on the ability to culture or isolate an organism^46^, although functionality is strongly dependent on the activity of the original promoter in the host cell and typically expression levels vary widely^47^. Previous functional metagenomics work has yielded many successes in identifying new enzymes^47^ as well as increasing tolerance to specific acute environmental stresses^48,49^.

To be truly useful, several key variables that play critical roles in functional metagenomic screening must be characterized. For example, for a given DNA source and host organism, the two fundamental variables in experimental design are fragment size and library copy number. These have not been explored systematically and it is not clear which values should be preferred. For instance, although larger inserts may contain intact clusters of related genes performing similar tasks^50^, they may also contain unrelated, deleterious sequences that impose a fitness cost^37,51^. Similarly, while it seems intuitive that more expression of a beneficial genetic tool further improves fitness, this may not be correct. Thus, a higher gene dosage may lead to benefits from higher gene expression, but may also disrupt the availability of some transcription factors to perform other functions or create a counterproductive metabolic load. Here, in an effort to increase the utility of functional metagenomics approaches, we looked at the importance of these foundational variables, insert size and copy number, and found that both can have outsized impacts on the efficacy of the introduced genetic material in non-intuitive ways.

## Results

### Creation and selection of functional metagenomic libraries

To explore these ideas, we created metagenomic libraries with varying insert sizes and studied the relationship between these key experimental parameters and their ability to increase fitness of the transformed *Escherichia coli* (strain Epi300 T1R). We used genomic DNA (gDNA) from 98 species: 96 described in a previous study of UV-resistant extremophiles^9^, the well-characterized polyextremophile *Deinococcus radiodurans*^10,52^, and the robust *E. coli* strain K12. Pooled gDNA was fragmented by sonication for varying amounts of time to yield a range of sizes, which were then separated by gel electrophoresis and cut from the resulting agarose gel in six size ranges (< 1 kb, 1–3 kb, 3–6 kb, 6–10 kb, 10–20 kb, and > 20 kb), and purified.

**Fig. 1:**
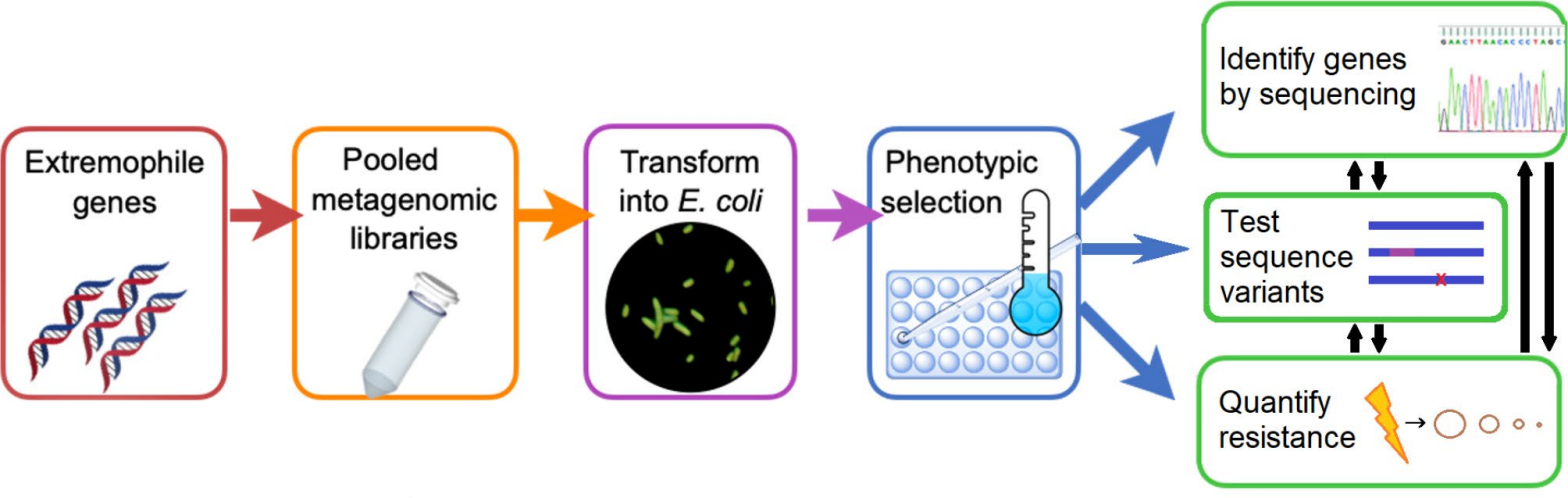
Experimental schematic of metagenomic libraries and screening.

The purified gDNA from different size ranges were cloned into the variable copy number pCC1FOS vector backbone and introduced to *E. coli* strain Epi300 T1R through viral transduction following the manufacturer’s instructions (Lucigen #CCFOS059, CopyControl™ HTP Fosmid Library Production Kit), yielding thousands of independent colonies on LB agar plates (see Methods). Colonies from each insert size range were resuspended and pooled in LB to begin the library screening assay (Fig. 1). Each library pool was exposed to 20 s UV-C radiation (2.65 W/m^2^ at 254 nm), estimated to kill 99.9% of *E. coli* with baseline tolerance. UV-C radiation was chosen for the focus of this study because it represents a novel environmental stress on present day Earth as the stratospheric ozone layer attenuates solar UV radiation below ~ 290 nM^27^ while also approximating the peak absorbance and thus vulnerability of nucleic acids and polypeptides^53^. This exposure was chosen to provide a large selective advantage to any cells containing an insert that increased UV resistance, while minimizing stochastic loss that could arise through overly harsh conditions. Survivors were allowed to recover for 24 h in fresh LB, and the process was repeated daily for 10 days. On the final day, survivors were plated on LB agar plates to yield the final UV-resistant isolates. Through this pooled and iterative selection, the different library entries compete directly against each, as increased UVR tolerance directly leads to increased representation in the heterogeneous culture. This facilitates identification of those conferring the strongest selective advantage rather than surveying for all constructs conferring advantage. We purified and transformed these selected constructs into new *E. coli* Epi300 T1R before functional testing to eliminate the potential for background genomic mutations that may have accumulated and also influence UV resistance.

### Functional testing of library isolates

**Fig. 2:**
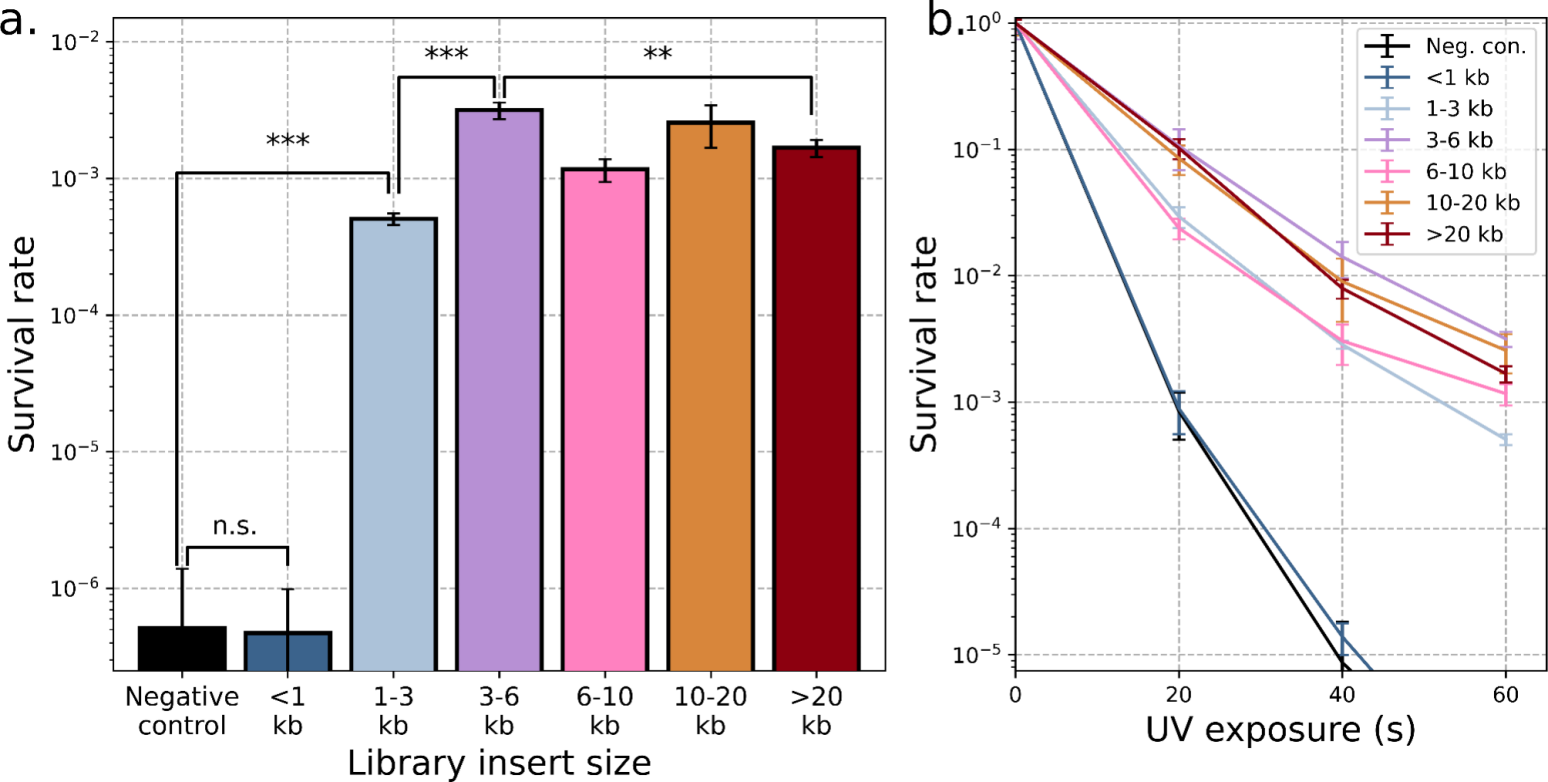
Increased UV tolerance from metagenomic library selection. (**a**) Survival after exposure to 2.65 W/m^2^ of UV exposure (254 nm) for 60 s for the most successful library entries in six different insert size ranges. The < 1 kb insert did not differ significantly from the negative control (*p* = 0.95). The 1–3 kb insert is significantly more UV-tolerant than the negative control (*p* = 2.11 × 10^− 6^) but significantly less UV-tolerant than the 3–6 kb insert (*p* = 4.25 × 10^− 5^). The 3–6 kb insert is also more UV-tolerant than the > 20 kb insert (*p* = 0.0021). (**b**) The constructs showed consistent differences in survival rate across a range of UV exposures. Error bars represent standard deviation across *n* = 3 replicates.

To examine the impact of library insert size, we tested the UV-C radiation resistance of cultures containing constructs from each size range. Confluent cultures were washed in phosphate buffered saline (1x PBS, Fisher Scientific BP2944-100) to remove UV-absorbing organics, exposed to varying amounts of UV radiation, and survival quantified through serial dilution and plating (see Methods). The smallest insert size range (< 1 kb) did not differ significantly from the negative control, but all larger inserts increased UV tolerance by > 1,000-fold (Fig. 2a). These trends were consistent across a range of UV radiation exposures (Fig. 2b). The smallest successful construct (1–3 kb size range) was significantly less UV-resistant than the larger constructs. This suggests it contained an effective but not complete resistance-conferring sequence. Although the largest insert sizes also underperformed relative to the strongest constructs (3–6 kb inserts), the loss of performance was far more severe for the undersized inserts.

### Sequencing of functional metagenomic library isolates

To better understand the cause of the increased tolerances, we purified plasmids from resistant isolates in each size class (ZymoPure™ Plasmid Miniprep Kit, Zymo Research #D4211) and sequenced them in their entirety (Elim Biopharmaceuticals, Hayward, CA USA). Several – but not all – constructs contained the archetypal DNA repair gene *recA*^54^. The gene *recA*, which is non-functional in Epi300 T1R *E. coli*, is not a surprising discovery and has been previously discovered in prior functional metagenomic screens for enhanced resistance to UV radiation^48^. This makes it an ideal positive control and test case to explore the importance of insert size, copy number, and genomic DNA origin in functional metagenomic analysis.

**Fig. 3:**
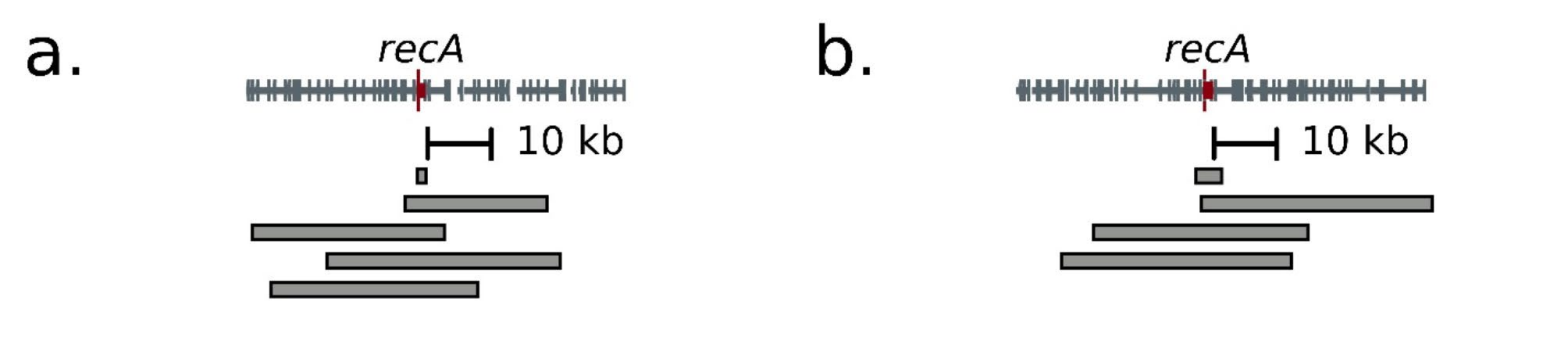
Diversity of *recA*-containing fragments recovered. The extent of fragments originating from strain K12 (**a**) and *P. agglomerans* (**b**) is denoted by horizontal bars under their respective genetic maps. The genetic maps denote the start of each new gene (horizontal grey bars) with a vertical bar and highlight *recA* in red.

Two distinct genetic sources in the functional metagenomics libraries yielded multiple independent isolates containing *recA* across a range of insert sizes: *E. coli* strain K12 and *Pantoea agglomerans* (formerly known as *Enterobacter agglomerans* or *Erwinia herbicola*), a plant-associated bacterium commonly found on flowers and trees that receive strong sunlight^55,56^ (Fig. 3). We observed similar increases in UV tolerance with sequences from both sources. Intriguingly, although the species are widely divergent across their genomes^57^, the *recA* coding sequences are 86% identical at the nucleotide level and 96% similar at the amino acid level, with the majority of differences being concentrated in the C-terminus. In comparison, the adjacent gene in both species, *recX*, does not align at the nucleotide level and is only 64% similar at the amino acid level. This may reflect more stringent sequence constraint at the *RecA* locus, recent horizontal gene transfer between the *E. coli* and *P. agglomerans* lineages, or experimental selection bias if only more similar sequences can function effectively in *E. coli*.

**Fig. 4:**
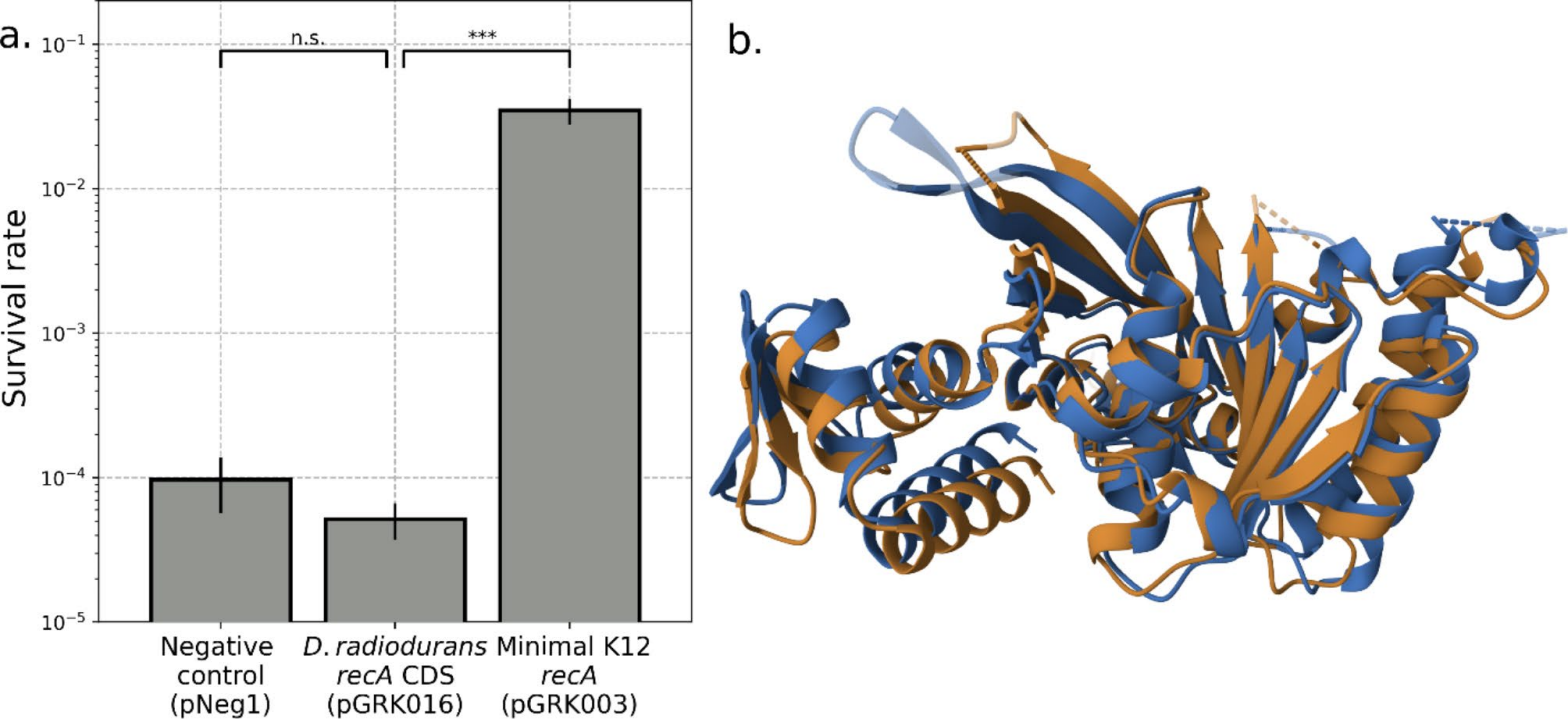
*D. radiodurans recA* does not increase UV tolerance. (**a**) *E. coli* transformed with either the *E. coli* strain K12 minimal construct (pGRK003), a variant of that construct with the coding sequence containing the same upstream promoter and downstream terminator sequences but with the *D. radiodurans recA* coding sequence (pGRK016), or a negative control plasmid (pNeg1) were exposed to 30 s of UV-C radiation (2.65 W/m^2^ at 254 nm) and survival quantified by spot dilution assay. The indicated p-values, from left, are 0.11 and 0.00014 by two-tailed t-test. Error bars represent standard deviation across *n* = 3 replicates. (**b**) Overlay of the RecA protein structures from *E. coli* strain K12 (blue) and from *D. radiodurans* (orange) shows high overall similarity as well as subtle differences, such as in α-helix phasing.

We also cloned and tested the *recA* gene from *Deinococcus radiodurans* and observed no effect on UV tolerance. The eponymous radiation resistance of *D. radiodurans* has been hypothesized to be a consequence of exceptional DNA repair enzymes, including RecA^58^. We did not recover any sequences from *D. radiodurans* in our functional metagenomic assay. We postulate that could be the consequence of differing gene expression mechanisms. Replacing the coding sequence of the K12 *recA* with *D. radiodurans recA*, while maintaining the K12 regulatory sequences, also resulted in UV tolerance indistinguishable from the negative control (Fig. 4a), despite the peptide sequences being 76% similar and having highly similar protein structures (RMSD = 1.83, TM-score = 0.92)^59–61^ (Fig. 4b). The large impact of these differences is consistent with prior mechanistic studies of the two homologs^62^ and highlights how homologous proteins that nominally perform the same functions may do so in different and incompatible ways. It is also possible that the recA protein from *D. radiodurans* did not express as expected, perhaps due to differences in GC content, codon bias, transcript stability, different chaperones, or other mechanisms that may limit genetic compatibility between some organisms.

Not all constructs with demonstrated radiation resistance contained *recA*. For example, one construct conferred almost the same increase in UVR tolerance while containing a completely unrelated 9.8 kb insert from K12. This sequence contains a cluster of genes involved in stress responses, including *yciT*^63,64^, *yciH*^65^, *osmB*^66^, *lapAB*^67,68^, *ribA*^69,70^, and *acnA*^71^. Previous work has highlighted the potential for stress response genes to play an important role in handling several distinct stressors. For example, the overexpression of exogenous *irrE* in *E. coli* increases both radioresistance^72^ and salt tolerance^43,73^, emphasizing that even genes primarily associated with osmotic stress, such as *osmB*, can facilitate increased radiation resistance. Future work to molecularly dissect this construct could definitively identify which gene or genes underlie the improved resistance to UV radiation. This highlights how clusters of adjacent genes can collectively yield a phenotype comparable to single key genes, and suggests that larger insert sizes are valuable to allow such discoveries. It also illustrates the richness and potential of genomic resources available even in closely related species or within species.

### Molecular dissection of UV resistance

**Fig. 5:**
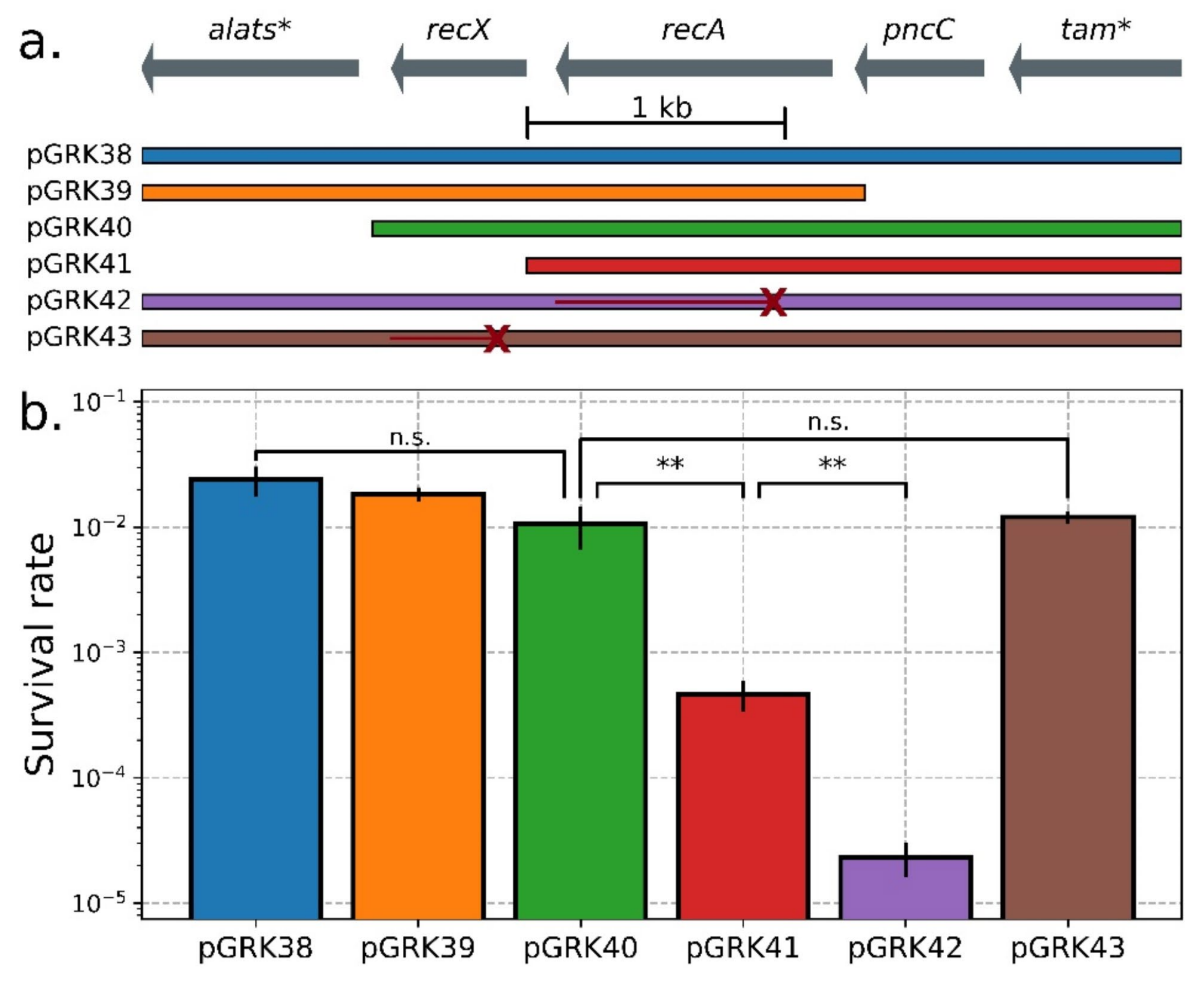
Molecular dissection of UV resistant insert from *Pantoea agglomerans*. (**a**) A genetic map of the metagenomic library sequence derived from *P. agglomerans* (top) and derived variants, either complete (pGRK38), with fragments deleted (pGRK39, pGRK40, pGRK41), or with frameshift mutations causing premature stop codons in *RecA* (pGRK42) or *RecX* (pGRK43). The genes on the ends, tam* and alats*, are incomplete fragments. (**b**) *E. coli* containing the constructs from (a) were exposed to 30 s UV radiation (2.65 W/m^2^ at 254 nm) and survival quantified by spot dilution assay. Significant differences were not observed between any fragments containing both *recA* and *recX* (*p* = 0.067), but the fragment lacking *recX* (pGRK41) had significantly lower survival (*p* = 0.023) though still higher than the nonfunctional *recA* (pGRK42, *p* = 0.0087). Restoring the *recX* sequence but not its activity (pGRK43) fully rescues this UV sensitivity and does not significantly differ from the intact sequence (*p* = 0.66). Error bars represent standard deviation across *n* = 3 replicates.

To understand the difference in UV resistance between the smallest and larger *recA*-containing inserts (Figs. 1, 2 and 3 kb vs. 3–6 kb), we created variants of the most resistant construct (pGRK38, 4045 bp insert from *P. agglomerans*) (Fig. 5). Deleting sequences upstream (pGRK39, orange) or downstream (pGRK40, green) of *recX* had no effect on UV resistance, but additionally deleting *recX* (pGRK41, red) significantly reduced UV resistance. Critically, this is not an effect of the recX protein, as the introduction of an early *recX* frameshift mutation causing a premature stop codon (pGRK43, brown) without deleting the nucleotide sequence fully rescues UV tolerance. Rather, the downstream *RecX* sequence must instead play a role in ensuring the correct expression of *recA*, such as through distal elements regulating transcription or modulating mRNA stability. Consistent with these ideas, *recX* has previously been shown to be co-transcribed with *recA*^74^. This suggests that it is not enough for a library insert to contain the causative gene and the adjacent intergenic regions, and that neighboring sequences may be important for maximizing functional gains independent of their coding status.

### Impact of expression level

**Fig. 6:**
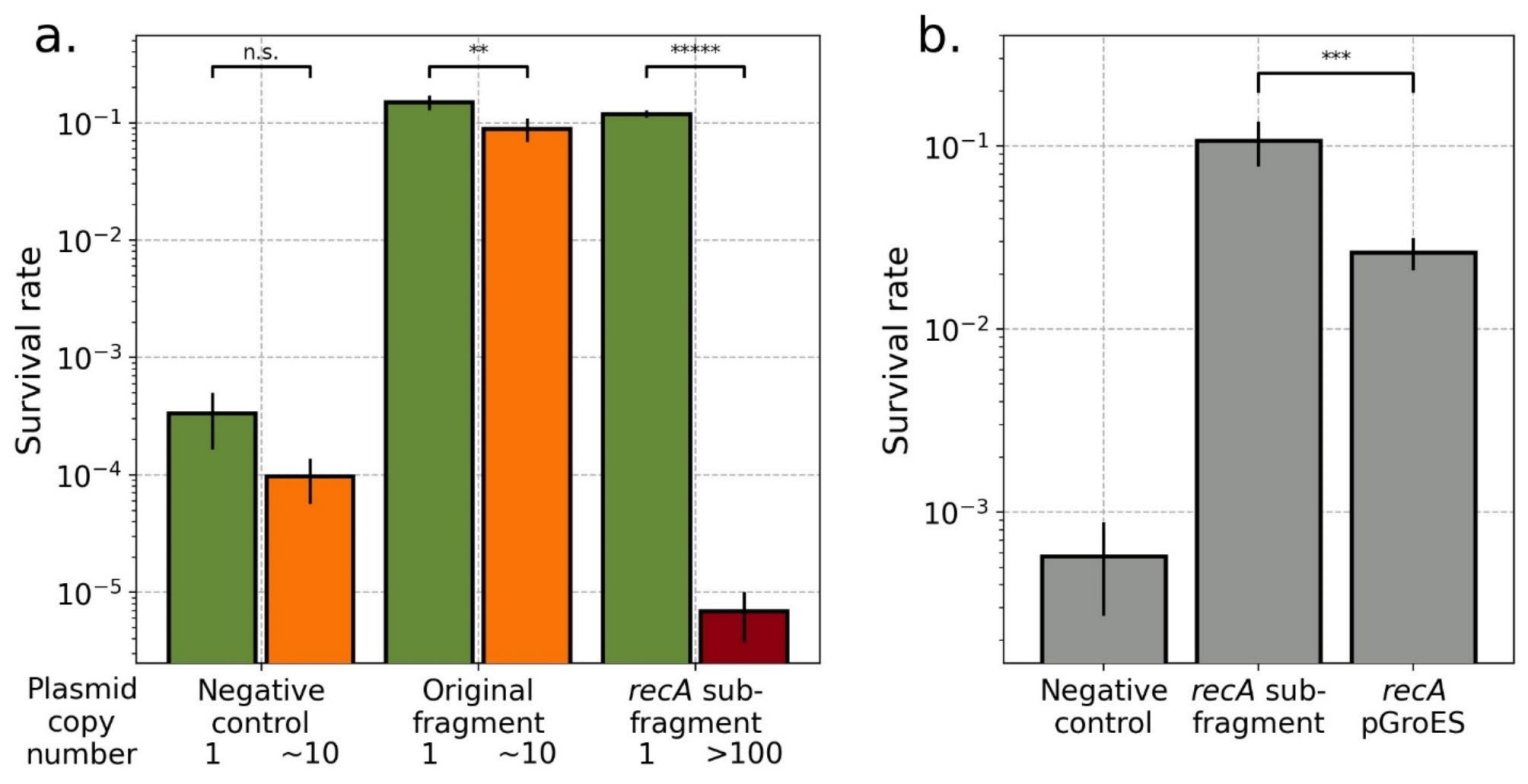
Survival does not scale with copy number or expression level. (**a**) *E. coli* were transformed with either a negative control plasmid (pNeg1, left), large 35 kb fragment containing *recA* and adjacent sequences (pGRK006, center), or smaller fragment containing only *recA* and its immediate promoter and terminator (pGRK003 and pGRK010, right) maintained at differing copy numbers (green low, orange medium, red high) and were exposed to 30 s of UV radiation (2.65 W/m^2^ at 254 nm) and survival was quantified by spot dilution assay. Survival was not affected by copy number in the negative control but was adversely affected in the two *recA* fragments (p-values, from left, are 0.057, 0.011, and 3.33e-7, by two-tailed t-test). (**b**) The *recA* promoter (from pGRK003, center) was replaced by the strong pGroES promoter (pGRK015, right) and samples were exposed to 20 s of UV radiation. Survival was decreased relative to the original promoter (*p* = 0.0036). For both, error bars represent standard deviation across *n* = 3 replicates.

We next explored the impact of plasmid copy number and gene dosage more generally to examine the hypothesis that adding more of a gene that increases UV resistance would lead to further increases in UV resistance. We utilized the variable copy number of the pCC1FOS plasmid backbone to test this, which can be induced to increase from single copy to > 10 copies per cell. However, contrary to our hypothesis, we found that increasing the copy number of the largest *recA*-containing fragment actually resulted in a small but significant decrease in UV resistance (Fig. 6a, middle). Increasing plasmid copy number had no effect on the negative control. Further, moving the core *recA* coding sequence and intergenic regions to the very high copy number pUC19 backbone (the whole *recA* and negative control fragments could not be moved to the high copy number backbone due to their large size) resulted in a dramatic decrease in UV resistance below even the negative control (Fig. 6a, right).

One possible explanation is that the increased copy number of the *recA* promoter could be binding transcription factors with multiple targets, thus reducing their availability to activate other DNA damage response genes. A second possible explanation is that the recA protein, which functions in conjunction with a large number of other proteins, including recF, recO, recR, dinI, recX, rdgC, psiB, uvrD, and ssb^75^, disrupts the stoichiometric balance of these other proteins when overexpressed, likewise reducing their effectiveness. To distinguish between these possibilities, we overexpressed RecA from a strong promoter (pGroES) at low plasmid copy number and observed (Fig. 6b) that UV resistance was diminished relative to the native promoter, but still significantly elevated compared to the negative control. This indicates that both proposed mechanisms play a role, but that the larger contribution is from copy number. This specific example illustrates the more general idea that adding a useful genetic tool may not be beneficial if added at the wrong dosage, and may even be detrimental.

## Discussion

As we explore the frontiers of biology beyond studying extant life into the novel challenges of astrobiology and bioindustrial applications, Theodosius Dobzhansky’s aphorism, “Nothing in biology makes sense except in the light of evolution”^76^ is more relevant than ever. To explore what is possible, whether optimizing production of biomolecules in the presence of challenging chemicals or evaluating the potential habitability of extraterrestrial environments, we need to develop methodology for efficiently expanding the environmental limits of life. This requires emulating as many of evolution’s innovations as possible and to reuse existing genetic tools in new applications. As evidenced by the tremendous resilience of some organisms to perchlorate^19,20^ and ionizing radiation^21–23^ levels exceeding any found on Earth by many orders of magnitude, even seemingly novel environmental challenges may have at least partial genetic solutions amongst Earth’s incredible biodiversity, if they can be found and redeployed.

The genetic tools developed by these organisms to address terrestrial environmental challenges are unlikely to be a complete solution for approaching the boundaries of life. For example, although salt export is a key adaptive strategy of many halophiles^77^, there are no known perchlorate export proteins, constituting a key gap in the genetic arsenal of life in the context of Martian perchlorate brines. In the future, *de novo* designed genes may be able to address these missing pieces and push the boundaries of life further still.

We have here built upon previous functional metagenomic studies to systematically explore the foundational variables of insert size and copy number and shown that they can have significant and counterintuitive effects. The specific size range that performed best here (3–6 kb) may not prove to be the ideal balance of opposing size concerns for all applications (for example, modulating membrane lipid composition may involve multiple sequential genes and benefit from a larger insert size), but rather illustrates how even a fragment containing the critical gene and flanking intergenic sequences may nonetheless dramatically underperform (Figs. 2 and 5).

On the other hand, additional increases in insert size do not yield further gains, and actually result in a small decrease in gained resistance. This could result from additional genes in the larger constructs that do not contribute to UV resistance performing activities that increase cellular stress either by their function or simply the metabolic load of increased insert size. This is consistent with prior studies that found selection against extraneous sequences in natural horizontal gene transfer^34,51,78^. However, while the oversized inserts (even those 10x larger) performed worse, it was a marginal difference, in sharp contrast with the more dramatic performance reduction of undersized inserts (Fig. 2). We therefore recommend erring on the side of larger inserts, which can subsequently be reduced in size as needed.

In contrast, higher copy number and higher expression can yield a markedly detrimental effect, sometimes even relative to the original, unmodified strain (Fig. 6). This defies the intuition that more of the correct genetic tool should yield further benefits and highlights the need for caution with these parameters. Importantly, this does not imply that low copy number backbones are superior, as previous functional metagenomics studies have obtained excellent results with high copy number plasmid backbones^49,79,80^, but rather that different specific insert sequences yield optimal performance under different conditions. Consequently, screening functional metagenomic libraries at a given copy number, whether high or low, may result in overlooking or misevaluating some entries. Therefore, libraries should be screened at multiple copy numbers to maximize thoroughness, though low copy number screening is particularly important for the eventuality of genome integration for enhanced stability. Similarly, cDNA-derived expression libraries, although not examined directly here, are likely to see great benefit from testing transcripts under a variety of promoters rather than a single strong promoter.

In nature, adaptations to environmental conditions are typically the result of multiple independent genetic changes^30,81,82^, and so to evolve life beyond its current capabilities, we should expect to likewise require structured exploration of steps beyond the work performed here and in other functional metagenomic studies of identifying individual genetic tools, regardless of their performance. One critical next step is to systematically test combinations of such tools for additive fitness gains. This may be done through intraculture recombination^83^, iterative plasmid construction, or iterative genomic integration. Likewise, even strongly selected genetic tools are unlikely to be fully optimized for either the intracellular environment of their new host or for the specific environmental challenge of interest. Thus, additional performance may be realized from these tools through subsequent regulatory and/or coding changes, either through traditional adaptive laboratory evolution or facilitated by an orthogonal replication system to more rapidly sample sequence variants^84^. Thus viewed in the light of natural evolutionary processes, organized raiding of nature’s genetic toolbox for individual tools is a critical first step of many to realize the common demands of both astrobiology and bioindustry to extend the limits of life.

## Methods

### Template DNA sources and purification

Glycerol stocks of 106 previously isolated UV-resistant microbes were thawed and grown for 72 h on agar plates as previously described^9^. Ninety six of the 106 described cultures grew, and only these were used in subsequent steps. Each culture was seeded into 100 µL of its respective liquid medium and grown for 48 h at 30 C. Samples were pooled in batches of 10, and purified by the NEB Monarch^®^ Genomic DNA Purification Kit (NEB #T3010S) according to the manufacturer’s instructions. DNA preparations from *Deinococcus radiodurans* strain R1 (ATCC 13939) and *E. coli* strain K12 were purified as controls. Each DNA preparation was quantified by Nanodrop and pooled equally by mass to yield a single final sample containing DNA from all 98 source cultures.

### Size selection

The pooled DNA sample was subjected to four different conditions: no treatment, 1 s sonication, 10 s sonication, or 45 s sonication [Sonicator Ultrasonic Processor XL2020, Misonix Inc, with microtip at level 4.5] to generate fragments at a range of sizes. The treated samples were run on a 0.7% low melt-agarose (Promega #V211) gel overnight at 20 V, and the desired insert band size ranges were cut from the gel with a razor, dissolved with GELase (Lucigen #E0032-1D), and purified by ethanol precipitation.

### Library construction

Functional metagenomic libraries were prepared from the size-selected DNA samples with the CopyControl™ HTP Fosmid Library Production Kit (Lucigen #CCFOS059), following manufacturer’s instructions, with adjustment of the insert and backbone mass during ligation to account for the differing insert sizes being tested. Following lentiviral transduction, cultures were plated on LB plates with 30 µg/mL chloramphenicol for amplification and quantification. Plates were then suspended in LB to begin repeated growth and UV exposure for selection of the pooled libraries, as described below. This yielded a total of 7,678 independent colonies, with more colonies from the larger insert sizes. Based on the expected insert sizes, these libraries span a total of 160 Mb (approximately 53 genomes averaging 3 Mb each), thus including roughly coverage of up to half of the total input DNA in the resultant metagenomic library. An additional library construction process with the same input DNA but fewer size categories yielded 70,950 colonies spanning an estimated 1259 Mb. Isolates from this second set of libraries yielded similar resistances and underlying sequences to those from the libraries described here.

### UV exposure

Confluent overnight cultures were collected by centrifugation at 5600 x g, resuspended in 1x PBS, then centrifuged and resuspended in PBS an additional time to remove organic matter from the growth media that may absorb UV radiation. Droplets containing 100 µL of culture in PBS were then placed in Petri dishes in a fixed location in a NU-425-400 biological safety cabinet and the germicidal UV light (peak emission at 254 nm) activated (for time depending on the specific experiment). Irradiance was measured with a Solar Light PMA2100 sensor fitted with a germicidal UVC sensor as 2.65 W/m^2^. Droplets were then collected and used for either continued UVR selection or quantification by dilution spot assay.

### UV radiation selection

Libraries were screened for entries that conferred increased resistance to UV radiation by daily UV exposure (as described above) and recovery periods for 10 days. After UV exposure, the samples were diluted in 3 mL LB with 30 µg/mL chloramphenicol for overnight recovery and growth. This process was then repeated for 10 days. After the final UV exposure, the samples were spread on LB agar plates to allow for picking and characterization of individual colonies.

### Quantifying survival by dilution spot assay

All reported survival rates derive from triplicate assays of each construct, both with and without UV exposure (for a total of six measurements per construct per condition). Cultures were prepared and either exposed or not exposed to UV radiation as described above. All samples were serially diluted by 10-fold and 10 µL droplets of each dilution were plated on LB agar plates and incubated at 37 °C overnight. The resulting colonies were counted at the lowest dilution with distinct colonies, and these counts were used to compute surviving cells in the undiluted sample. Survival rates were calculated by dividing the surviving cell count for each of the UV-exposed samples by the average cell count of the unexposed samples.

### Copy number induction

Plasmid pCC1FOS was induced to high copy number by the addition of Lucigen CopyControl Induction Solution (Lucigen #CCIS125) and incubation at 37 °C with vigorous shaking for 24 h, following the manufacturer’s instructions.

### Molecular cloning

Variants of the successful library entries were made following standard molecular biology techniques. Briefly, fragments were PCR amplified with Q5 polymerase (NEB #M0492S) with tagged primers (detailed primer and plasmid construction tables below). Amplicons were visualized on an agarose gel (0710-500G, VWR Life Sciences) stained with 0.5 ug/mL ethidium bromide by an Azure 200 Gel Imager (Azure Biosystems). Residual template was digested by DpnI (NEB #R0176L) and products column purified (Zymo Research DNA Clean & Concentrator, #D4004). When homology-based cloning methods were used, fragments were then combined through HiFi DNA Assembly (NEB #M5520AA) and transformed into chemically competent Epi300 T1R *E. coli*. When restriction digest-based cloning methods were used, DNA was digested with BamHI-HF (NEB #R3136L) and SacI-HF (NEB #R3156L) in 1x rCutSmart buffer (NEB #B6004S), products gel-purified (Zymo Research Zymoclean Gel DNA Recovery Kit, #D4002, and ligated with T4 DNA Ligase (NEB #M0202L) and transformed into chemically competent Epi300 T1R *E. coli.* The resulting colonies were picked, grown overnight in LB with chloramphenicol (30 µg/mL, Sigma #C0378) or carbenicillin (50 ug/mL, Fisher Scientific #BP26481), plasmid DNA extracted and purified (Zymo Research Plasmid Miniprep Kit #D4211), and sequenced by Elim Biopharmaceuticals. Further details on the construction of all genetic constructs are given in Supplemental Table S1 and Supplemental Table S2. The Supplemental Materials also contain their full sequences.

### Statistical methods

All error bars represent standard deviation. All statistical tests are performed as unpaired, two-sided Student’s t-test. Unless otherwise noted, all reflect results of *n* = 3 replicates.

## Electronic supplementary material

Below is the link to the electronic supplementary material.

Supplementary Material 1

## Data Availability

Sequences of all genetic constructs have been deposited in GenBank with accession numbers PQ202963-PQ202979 (additional details in Table S3). Physical data in the form of purified plasmids and bacterial strains will be stored in the Rothschild Lab for at least 5 years and available upon request to Dr. Rothschild.
