# Supplementary material for: Raiding nature’s genetic toolbox for UV-C resistance by functional metagenomics
Source: Sci Rep. 2025 Jan 2;15:223. doi: 10.1038/s41598-024-83952-w (PMC11695868; doi:10.1038/s41598-024-83952-w)
Supplement: Supplementary file 1 — Supplementary Material 1 [file 41598_2024_83952_MOESM1_ESM.docx]

Supplemental Materials

for

Raiding nature’s genetic toolbox for UV-C resistance by functional metagenomics

Garrett A. Roberts Kingman, Justin L. Kipness, and Lynn J. Rothschild

Corresponding author: Lynn J. Rothschild (lynn.j.rothschild@nasa.gov)

**Table S1: Plasmid construction**

| **Plasmid name** | **Description** | **Backbone source** | **Insert source and assembly method** |
| --- | --- | --- | --- |
| pNeg1 | Negative control plasmid | Fosmid Library Production Kit (Lucigen #CCFOS059) positive control provided by manufacturer. Contains 42 kb insert with no expected activity. Used as negative control in UV assays. | |
| pGRK003 | Minimal *recA* sequence from strain K12 | Metagenomic library screening in pCC1FOS | |
| pGRK010 | pGRK003 insert in high copy number plasmid | Digest pUC19 with BamHI and SacI | Amplify pGRK003 with oGRK060/oGRK061, digest BamHI and SacI, purify, and ligate |
| pGRK015 | pGRK003 with pGroES promoter replacing endogenous promoter | Amplify pGRK003 with oGRK074/oGRK075 | No separate insert. Circularize backbone fragment using HiFi assembly |
| pGRK016 | pGRK003 with CDS of *recA* from *D. radiodurans* replacing CDS of *recA* from strain K12 | Amplify pGRK003 with oGRK072/oGRK073 | Amplify *D. radiodurans* gDNA with oGRK070/oGRK071, purify, and use HiFi assembly |
| pGRK038 | 4045 bp RecA sequence from *P. agglomerans* | Metagenomic library screening in pCC1FOS | |
| pGRK039 | pGRK038 with deletion of genes upstream of *recA* | Digest pCC1FOS with BamHI | Amplify pGRK038 with oGRK168/oGRK169, purify, and use HiFi assembly |
| pGRK040 | pGRK038 with deletion of genes downstream of *recX* | Digest pCC1FOS with BamHI | Amplify pGRK038 with oGRK167/oGRK170, purify, and use HiFi assembly |
| pGRK041 | pGRK038 with deletion of genes downstream of *recA* | Digest pCC1FOS with BamHI | Amplify pGRK038 with oGRK167/oGRK171, purify, and use HiFi assembly |
| pGRK042 | pGRK038 with introduced frameshift mutation in *recA* leading to premature stop codon | Amplify pGRK038 with oGRK172/oGRK036 | Amplify pGRK038 with oGRK173/oGRK012, purify, and use HiFi assembly |
| pGRK043 | pGRK038 with introduced frameshift mutation in *recX* leading to premature stop codon | Amplify pGRK038 with oGRK174/oGRK036 | Amplify pGRK038 with oGRK175/oGRK012, purify, and use HiFi assembly |

**Table S2: Sequences of primers used in plasmid construction**

| **Name** | **Sequence** |
| --- | --- |
| oGRK012 | GTAAAACGACGGCCAG |
| oGRK036 | CTGGCCGTCGTTTTAC |
| oGRK060 | TCTAGAGGATCCGTATGCATTGCAGACCTT |
| oGRK061 | GAATTCGAGCTCGATGCGACCCTTGTGTA |
| oGRK070 | ATGAGCAAGGACGCCACC |
| oGRK071 | TTACGCTTCGGCGGCTTC |
| oGRK072 | TTGGTGGCGTCCTTGCTCATttttactcctgtcatgc |
| oGRK073 | CCGAAGCCGCCGAAGCGTAAtcgtcttgtttgatacacaag |
| oGRK074 | CATTTCACTTTTCAAGGAGCAATCACAatggctatcgacgaaaacaaac |
| oGRK075 | ATTGCTCCTTGAAAAGTGAAATGAGGAAGAttttactcctgtcatgcc |
| oGRK167 | AGGTCGACTCTAGAGGATCCCACatctgggatgcgggcccggt |
| oGRK168 | CGGTACCCGGGGATCCCAcgatttgttactgaggtcagtggcacc |
| oGRK169 | AGGTCGACTCTAGAGGATCcctgcagaaactgtatggcgaatttc |
| oGRK170 | AATTCGAGCTCGGTACCCGGGgtggtgcctgattagcggaacaaaaag |
| oGRK171 | CGAATTCGAGCTCGGTACCCGGGgcgcaccttgtacaaacagagg |
| oGRK172 | ctgcaaagtcacgtggtttt |
| oGRK173 | aaaaccacgtgactttgcag |
| oGRK174 | gttgcagtttaCcgcgctaac |
| oGRK175 | gttagcgcgGtaaactgcaac |

**Table S3: Description of sequences and GenBank accession numbers**

| **Name** | **Description** | **GenBank accession number** |
| --- | --- | --- |
| eGRK168_4C | UV-resistant isolate from functional metagenomic screening. 9.9 kb insert from *E. coli* strain K12. Contains 9 complete genes (no *recA*). | PQ202963 |
| eGRK168_5A | UV-resistant isolate from functional metagenomic screening. 22.3 kb insert from *E. coli* strain K12. Contains *recA*. | PQ202964 |
| eGRK168_6C | UV-resistant isolate from functional metagenomic screening. 36.9 kb insert from *E. coli* strain K12. Contains *recA*. | PQ202965 |
| eGRK168_I1 | UV-resistant isolate from functional metagenomic screening. 36.8 kb insert from *P. agglomerans*. Contains *recA*. | PQ202966 |
| eGRK168_I5 | UV-resistant isolate from functional metagenomic screening. 34.3 kb insert from *P. agglomerans*. Contains r*ecA*. | PQ202967 |
| eGRK168_LA | UV-resistant isolate from functional metagenomic screening. 32.8 kb insert from *E. coli* strain K12. Contains *recA*. | PQ202968 |
| eGRK168_LC | UV-resistant isolate from functional metagenomic screening. 36.7 kb insert from *P. agglomerans*. Contains *recA*. | PQ202969 |
| pGRK003 | UV-resistant isolate from functional metagenomic screening. 1.2 kb insert from *E. coli* strain K12. Contains *recA*. | PQ202970 |
| pGRK010 | Insert from pGRK003 in high copy number plasmid backbone (from pUC19) | PQ202971 |
| pGRK015 | pGRK003 with original promoter replaced by constitutive pGroES promoter | PQ202972 |
| pGRK016 | pGRK003 with original *recA* CDS replaced by *recA* CDS from *D. radiodurans* | PQ202973 |
| pGRK038 | UV-resistant isolate from functional metagenomic screening. 4.0 kb insert from *P. agglomerans*. Contains *recA* (see Fig. 5a). | PQ202974 |
| pGRK039 | pGRK038 with deletion of genes upstream of *recA* (see Figure 5a). | PQ202975 |
| pGRK040 | pGRK038 with deletion of genes downstream of *recX* (see Figure 5a). | PQ202976 |
| pGRK041 | pGRK038 with deletion of genes downstream of *recA* (see Figure 5a). | PQ202977 |
| pGRK042 | pGRK038 with introduced frameshift mutation in *recA* leading to premature stop codon (see Figure 5a). | PQ202978 |
| pGRK043 | pGRK038 with introduced frameshift mutation in *recX* leading to premature stop codon (see Figure 5a). | PQ202979 |
